# High-Intensity Continuous Light from Red–Blue Light-Emitting Diodes Improved Yield, Nutritional Quality and Reactive Oxygen Species Accumulation in Two Leaf-Color Lettuces

**DOI:** 10.3390/biology13121077

**Published:** 2024-12-20

**Authors:** Wenke Liu, Bing Liu, Qibao Wu

**Affiliations:** 1 Institute of Environment and Sustainable Development in Agriculture, Chinese Academy of Agricultural Sciences, Beijing 100081, China; liuwenke@caas.cn; 2 School of Traffic and Environment, Shenzhen Institute of Information Technology, Shenzhen 518172, China; liubing0708@sziit.edu.cn; 3 School of Intelligent Manufacturing and Equipment, Shenzhen Institute of Information Technology, Shenzhen 518172, China

**Keywords:** light intensity, nutritional quality, antioxidant substance, reactive oxygen species, DPPH

## Abstract

In an environmentally controlled plant factory with LED red–blue light, the effects of conventional light (4R:1B, 200 μmol·m^−2^·s^−1^, 18/6 h) and continuous light (CL, 24/0 h) with three light intensities (4R:1B, 200, 300 and 400 μmol·m^−2^·s^−1^, 24/0 h) on yield, nutritional quality, reactive oxygen species (ROS) content and 1,1-diphenyl-2-picrylhydrazyl radical scavenging activity (DPPH) in green-leaf Yidali and purple-leaf Zishan lettuces were investigated. The results showed that the dry and fresh shoot weight of two lettuces exposed to CL tended to increase with light intensity—from 200 to 400 μmol·m^−2^·s^−1^—compared to conventional light, while the leaf area tended to decrease or remained unchanged. High-intensity CL could significantly increase soluble sugar and reduce the nitrate contents of the two lettuces. Also, the antioxidant substance (anthocyanins, flavonoids and total phenols) content of the two lettuces was improved with the increase in CL intensity. High-intensity CL could significantly increase the malondialdehyde, hydrogen peroxide and superoxide anion content and DPPH of the two lettuces. The above indices showed similar results both at 6 and 12 days after light treatment. In contrast, the Zishan cultivar contained more antioxidant substances, ROS and MDA contents and DPPH (more than 1 to 100 times) than the Yidali cultivar under high-intensity CL. In summary, high-intensity CL could improve the yield and nutritional value of both Yidali and Zishan lettuces. The high CL tolerance of Zishan was attributed to a stronger antioxidant capacity due to a greater content of antioxidant substances and DPPH, while the accumulation of ROS and the content of antioxidant substances might interact.

## Simple Summary

Light intensity is an important factor that impacts the continuous light (CL) effects of LEDs’ red–blue light on nutritional quality and reactive oxygen species (ROS) accumulation in two leaf-color lettuces. In this study, the effects of conventional light and CL with three light intensities on yield, nutritional quality, ROS content and 1,1-diphenyl-2-picrylhydrazyl radical scavenging activity (DPPH) in green-leaf Yidali and purple-leaf Zishan lettuces were investigated. We found that high-intensity CL could improve the yield and nutritional value of both Yidali and Zishan lettuces. The high CL tolerance of Zishan was attributed to stronger antioxidant capacity due to a greater content of antioxidant substances and DPPH, while the accumulation of ROS and the content of antioxidant substances might interact.

## 1. Introduction

The potential of LED red–blue continuous light (CL) to promote growth and yield in facility horticulture concerns international scholars [1,2,3,4,5], especially for plant factories with artificial light (PFALs) [6,7,8]. It had been shown that LED red–blue CL could improve the productivity of hydroponic lettuces in PFALs [6,7]. In fact, CL is also a kind of light stress as it eliminates dark periods. Based on the sensitivity of crops to CL, horticultural plants can be divided into two categories, i.e., sensitive plants (tomato, cucumber, *Allium fistulosum* onion and green-leaf lettuce etc.) and tolerant plants (pepper, *Allium cepa* onion and purple-leaf lettuce etc.) [1,5,6]. The majority of horticultural plants belong to sensitive plant species, and their yield is usually decreased and the leaves damaged under CL via negative photo-oxidation effects on the photosynthesis system [1,5,6]. Moreover, cultivars of the same plant species differed largely and can even have an opposite capacity to tolerate CL [1,5]. In a previous study, it was found that the yields of Yidali and Zishan lettuces were increased significantly under LED red–blue CL, but their tolerance to CL was very different. CL induced leaf damage in the Yidali lettuce, while the Zishan lettuce remained normal [6]. Moreover, the CL tolerance of Yidali lettuce was regulated by light intensity, light quality and duration [4,7,8].

Lettuce (*Lactuca sativa*) is a global leafy vegetable that is widely grown and consumed, rich in many nutrients. Due to its diverse varieties and fast growth rate, it is particularly suitable for large-scale production in PFALs, and it has been regarded as a model plant for photobiology research in PFALs [9,10]. However, in actual production, low-quality traits (high nitrate content, low vitamin C and soluble sugar contents etc.) and the use of high-dose nitrate fertilizer for hydroponically grown leafy vegetables in PFALs have been crucial problems [11,12,13,14,15]. In recent years, more research about nutritional quality-improvement technology for hydroponic lettuce has been attempted using LED light environment control, such as 24 h lighting using red and blue light in PFALs [7,10,11]. CL is a special light pattern that usually exists only in horticultural facilities with artificial light, such as PFALs and modern greenhouses. CL breaks the circadian rhythm of natural and conventional light, providing plants with 24 h lighting, maximizing the time of light and photosynthesis, and eliminating the dark period and respiratory depletion during the dark period. Thus, CL has the potential to promote growth and yield for facility plants.

In recent years, studies on CL have mainly focused on the effects of red–blue CL from LED light sources on plant growth, yield and quality, including CL trait factors such as light intensity, light quality, and duration and the alternating effects of CL light quality and intensity [8,15,16,17,18,19]. It had been shown that CL exposure of lettuces at a certain intensity could significantly increase the biomass of hydroponically grown leafy vegetables, and CL also significantly improved the quality of lettuces [7,13]. Zhou et al., (2012) [14] found that short-term red–blue CL before harvest significantly reduced nitrate content and increased the content of nutrients such as soluble sugar in hydroponically grown lettuces. Actually, CL is a kind of light stress compared with conventional light—if plants absorb too much light for photosynthesis under CL, this results in excess light energy and damage. An excess accumulation of excitation energy in the light energy system could cause the photoinhibition of photosynthesis and produce reactive oxygen species (ROS) [20]. To scavenge ROS formed under light stresses, researchers have increased the concentration of antioxidants in plants and observed the impacts [21,22]. Antioxidants in plants that are known to confer human health-promoting benefits acted as a part of the defense mechanism developed during the long-term evolution of plants to resist oxidative stress. However, if the light intensity was too high, plant defense mechanisms could not remove these ROS in time, which would lead to photo-oxidation and photodamage. To date, no study has been conducted to investigate the relationship between the nutritional quality and ROS accumulation of sensitive and tolerant lettuce cultivars under CL, which is important for revealing the physiological differences between CL-sensitive and CL-tolerant lettuce cultivars when exposed to long-term CL. Based on the damage mechanisms of CL and their impacts on plants, tolerant plant species or cultivars might have strong antioxidant capacities that enable them to remove the ROS produced by carbohydrate accumulation and circadian rhythm disorder [19]. This means that the capacity to control ROS content is the key factor that determines plant CL tolerance.

The light intensity of CL affected plant growth, quality and oxidative stress to different extents by enhancing photosynthesis or aggravating photo-oxidation. Zha et al. (2019a) [4] found that the content of hydrogen peroxide, superoxide anions and MDA in Yidali lettuce showed an increasing trend with light intensity after 12 days of CL treatment, up to the highest contents at 300 μmol·m^−2^·s^−1^. Studies had shown that phenolic acids, flavonoids, anthocyanins and other antioxidants have a strong ability to scavenge hydrogen peroxide, superoxide anions and hydroxyl radicals, which could interrupt the oxidative damage caused by ROS and free radicals, so as to achieve the purpose of an antioxidant. Moreover, the concentration of flavonoids was positively correlated with DPPH. Furthermore, photo-oxidative stress induced by CL could be attributed to excessive daily light integrals [4]. Zhou et al. (2012) [13] showed that the decrease in nitrate concentration and the increases in soluble sugars and AsA contents were relatively low at a CL light intensity of 50 μmol·m^−2^·s^−1^, but this increased gradually as the light intensity increased from 50 μmol·m^−2^·s^−1^ to 200 μmol·m^−2^·s^−1^. However, the marginal benefit of increased CL intensity in lowering nitrate concentration and increasing AsA content declined rapidly when the light intensity increased beyond 100 to 200 μmol·m^−2^·s^−1^. In view of the desire for improved human health and nutrition, high-quality leafy vegetables containing health-promoting substances with low nitrate content and high levels of soluble sugar are urgently needed, primarily for production in plant factories [13]. At present, no study on the effects of LED red–blue CL intensity on the yield and nutritional quality of CL-tolerant and -sensitive lettuce cultivars in PFALs has been published.

It is an urgent issue to investigate the physiological differences between CL-sensitive Yidali and CL-tolerant Zishan lettuces under CL with wider range of light intensity. Based on previous studies on the effects of LED red–blue CL intensity (100–300 μmol·m^−2^·s^−1^) on lettuce growth, the factors of AsA accumulation and metabolism [7,8], elevated CL light intensity (200–400 μmol·m^−2^·s^−1^) and conventional light (light intensity 200 μmol·m^−2^·s^−1^, red/blue ratio 4:1, photoperiod 18/6 h) were adopted to investigate differences in the quality, ROS, MDA, DPPH and content of antioxidant substances of two lettuce varieties—CL-sensitive Yidali and CL-tolerant Zishan lettuces—in a controlled environment. Through two-time sampling and indicator determination after 6 and 12 days of light treatment, the objectives are to clarify the differences of CL-sensitive Yidali and CL-tolerant Zishan lettuce cultivars in terms of yield, nutritional quality and ROS accumulation under a wider light-intensity range (200–400 μmol·m^−2^·s^−1^). The aim is to reveal physiological differences between CL-sensitive Yidali and CL-tolerant Zishan lettuces under CL with a wider range of light intensity.

## 2. Materials and Methods

### 2.1. Plant Materials and Growth Conditions

The experiment was conducted in the plant factory of the Institute of Environment and Sustainable Development in Agriculture, Chinese Academy of Agricultural Sciences, from November 2020 to December 2020. Yidali and Zishan lettuces were selected as experimental materials. On 17 November 2020, the seeds were planted in wet-sponge seedling blocks (2.5 cm × 2.5 cm × 2.5 cm) with an upper opening and placed in a dark environment for 2 days. The sponge blocks were bought from Beijing Haikehongchuang Biotechnology Co. in China. After the seeds germinated, on November 19, the seedlings were transferred to an environment with a light intensity of 200 μmol·m^−2^·s^−1^, and the photoperiod was 16/8 h, involving a white LED lamp (300 W, continuously spectral ranging blue light to red light, with the same size and shape). After 14 days of seedling growth, on 5 December, the seedlings were transplanted to a circulation hydroponic culture system. The circulation hydroponic culture system consisted of four layers of cultivation shelves, a nutrient solution circulation system, LED light panels, and an automatic control system. During the experiment, the temperature and humidity were maintained at 23 ± 1/20 ± 1 °C and 50–60%, respectively. During the experiment, the nutrient solution was hydroponic, and Hoagland nutrient solution was selected. An LED red–blue combination lamp board (Wuxi Huazhaohong Optoelectronics Technology Co., Ltd., Wuxi, China) was selected for illumination processing. The power of the LED light panel was 300 W. The size of the lamp board was 50 cm × 50 cm, the wavelength of the red light was 655 nm and the wavelength of the blue light was 437 nm. The lamp board was hung 45 cm above the cultivation tank. An LI-1500 irradiance measuring instrument (LI-COR, Lincoln, NE, USA) was used to measure the light intensity 5 cm above the center of the cultivation tank, and we adjusted the light quality and light intensity to a set value.

### 2.2. Light Treatments

**Figure 1:**
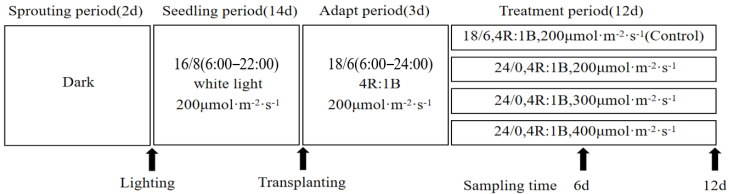
Schematic diagram of plant growth stages, experimental design and sampling time of LED red–blue CL intensity on yield, nutritional quality and reactive oxygen species accumulation in two leaf-color lettuces.

In order to adapt the lettuce seedlings to the nutrient-solution circulation culture system, they were transplanted under uniform light conditions (4R:1B, 200 μmol·m^−2^·s^−1^, 18/6 h) for 3 days before CL treatment. The ratio of red to blue light was 4:1, the photoperiod was 18/6 h, and the light intensity was 200 μmol·m^−2^·s^−1^. Two varieties of lettuce were subjected to three light treatments with a red/blue light ratio of 4:1 under CL conditions. The continuous illumination intensities were 200, 300 and 400 μmol·m^−2^·s^−1^, respectively. The light treatments in the seedling and treatment stages are shown in Figure 1.

### 2.3. Measurement Items and Methods

Samples were taken at the end of the sixth (13 December) and twelfth (19 December) day of CL treatment; three plants for each treatment were sampled each time. Then, the leaves (without petiole) were rapidly frozen with liquid nitrogen. The plant samples frozen with liquid nitrogen were ground into powder with a high-throughput tissue grinder at low temperature and stored in a refrigerator at −80 °C for future use.

The content of MDA was determined by the method proposed by Yang et al. (2010) [23]. After grinding 0.1 g frozen sample with liquid nitrogen, we added 1 mL of pre-cooled 10% tri-CL acetic acid (TCA) and centrifuged at 15,000 r for 10 min at 4 °C. We mixed 0.5 mL of supernatant and 0.5 mL of 0.6% thiobarbituric acid and boiled at 100 °C for 20 min, which we then quickly cooled to room temperature. After the reaction mixture was centrifuged at 15,000 r for 10 min, the absorbance of the supernatant at 450, 532 and 600 nm was measured to calculate the MDA content.

The H_2_O_2_ content was determined by the method of Brennan and Frenkel (1977) [24] using a detection kit (Beijing Solarbio Science & Technology Co., Ltd., Beijing, China). A total of 0.1 g frozen fresh sample was weighed and ground into powder in liquid nitrogen. Then, 1 mL pre-cooled acetone was used to extract H_2_O_2_. The homogenate was centrifuged at 10,000 r for 20 min at 4 °C. After centrifugation, we drew the supernatant, added 0.2 mL concentrated ammonia and 0.1 mL 10% (*v*/*v*) titanium tetrachloride-hydrochloric acid solution, mixed them immediately, and then centrifuged at 4000 r for 10 min at 25 °C. After discarding the supernatant, 1 mL acetone was added to wash off the pigment in the pellet. The mixture was then centrifuged again (25 °C, 4000 r, 10 min). We discarded the supernatant, dissolved the precipitate with 1 mL H_2_SO_4_, and measured the absorbance of the solution at 412 nm.

The superoxide anion content was measured by the method of Hodges et al. (1999) [25]. A total of 0.1 g of fresh samples from the mature lettuce leaves was ground and extracted with nitro blue tetrazolium (NBT) solution and subsequently filtered. We added nitro blue tetrazole (NBT) solution to the supernatant and mixed well, before boiling in water for 20 min. The absorbance of the extraction at 220 and 275 nm was measured with a spectrophotometer (Shimadzu TU-1800, Kyoto, Japan), respectively.

Soluble sugar was measured by the phenol-sulfuric acid method of Dubois et al. (1956) [26]. We took 0.1 g of fresh plant sample powder, added 1.5 mL of distilled water, and placed it in a boiling water bath for extraction. Then, we drew 0.5 mL of the sample solution into a test tube, added 1.5 mL of distilled water, added phenolic and concentrated sulfuric acid solution, and then measured and calculated the soluble sugar content. Then, the absorbance of the extraction at 485 nm was measured.

The nitrate content was determined by the sulfuric acid–salicylic acid method (Cataldo 1975) [27]. We took 0.1 g of fresh plant sample powder, added 1.5 mL of distilled water, and placed it in a boiling water bath for extraction. We took 0.1 mL of the extract into a 10 mL test tube, added 0.4 mL of 5% salicylic acid–concentrated sulfuric acid solution, mixed and cooled. Then, we added 9.5 mL of 8% sodium hydroxide solution and measured and calculated the content of nitrate after cooling.

Anthocyanin content was determined according to the pH differential method of Giusti and Wrolstad (2001) [28]. A total of 0.1 g of fresh samples from the mature lettuce leaves was ground and extracted with HCl-methanol and subsequently filtered. The homogenate was centrifuged (13,000 r, 15 min, 4 °C), and the supernatant was collected for the next steps. We took two copies of the supernatant—one with potassium chloride buffer, pH 1.0, and the other with sodium acetate buffer, pH 4.5. We allowed these dilutions equilibrate for 15 min, then measured the absorbance of each dilution at 530 and 700 nm against a blank cell filled with distilled water. With the same extraction method, the relative content of flavonoids could be determined by measuring the absorbance of the extract at 325 nm.

The total phenolic content was determined by using the Folin–Ciocalteu assay, following Khanam et al. (2012) [29]. A total of 0.1 g of fresh samples from the mature lettuce leaves were ground and extracted with 1 mL 80% aqueous methanol in an ultrasonic bath for 20 min. The homogenate was subsequently centrifuged (13,000 r, 15 min, 4 °C) and the supernatant was collected for the next steps. The absorbance of the extraction at 750 nm was measured by a spectrophotometer, and gallic acid was used as the standard reference. GAE was expressed as μg gallic acid equivalent (GAE)/100 g fresh weight.

DPPH was measured according to the method of Coruh et al. (2007) [30]. First, 0.01 g DPPH was dissolved in 100 mL absolute ethanol, and 40 mL of the prepared solution was added to 60 mL absolute ethanol to form a 0.1 mmol/L test solution (now used and prepared). Then, 0.1 g of sample was added to 1 mL absolute ethanol, homogenized in an ice bath, and centrifuged (4 °C, 10,000 r, 10 min), and then 40 μL supernatant and 400 μL detection solution were taken. Then, all the reaction mixture was kept away from light for 20 min. The absorbance at 517 nm was measured.

### 2.4. Statistical Analysis

Data were analyzed with SPSS 18.0 (SPSS, Inc., Chicago, IL, USA). Data were subjected to one-way analysis of variance (ANOVA), and significant differences among treatment means were determined using Duncan’s multiple range test at a 95% confidence level.

## 3. Results

### 3.1. Effects of LED Red–Blue CL Intensity on Biomass and Leaf Area of Two Lettuce Varieties

As shown in Table 1, the shoot dry and fresh weight of two lettuces exposed to CL tended to increase with a light intensity from 200 to 400 μmol·m^−2^·s^−1^ compared to conventional light at two sampling times, and the highest yield was obtained at 300 or 400 μmol·m^−2^·s^−1^. However, the leaf area of two lettuces under CL tended to decrease or remain unchanged with a light intensity from 200 to 400 μmol·m^−2^·s^−1^ compared to conventional light at two sampling times, and the lowest values were obtained at 300 or 400 μmol·m^−2^·s^−1^.

### 3.2. Effects of LED Red–Blue CL Intensity on Soluble Sugar and Nitrate Contents of Two Lettuce Varieties

**Figure 2:**
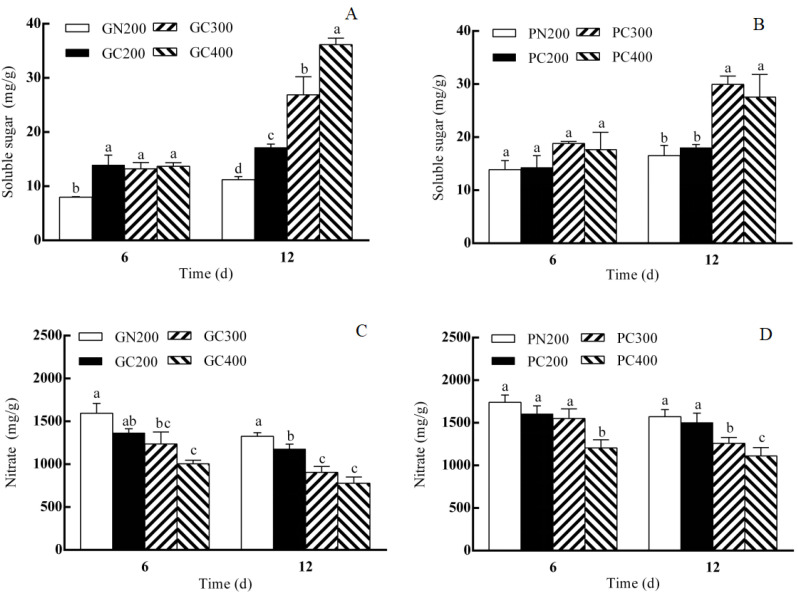
Effect of LED red–blue CL intensities on soluble sugar and nitrate contents in Yidali (**A**,**C**) and Zishan (**B**,**D**) lettuces after 6 and 12 days of treatment. Values and bars represent the means of three replicates ± SD. Within a time point, different letters indicate significant differences at the *p* < 0.05 level (Duncan’s multiple range test) over 6 and 12 days of separate tests.

As shown in Figure 2A, the soluble sugar content of Yidali lettuce increased after 6 days of treatment compared to the conventional light treatment, and there was no change in the soluble sugar content of Yidali lettuce with the increase in light intensity. The soluble sugar content of Yidali lettuce increased after 12 days compared with the conventional light treatment, and the soluble sugar content of Yidali lettuce increased with the increase in light intensity. The soluble sugar content of Zishan lettuce showed no significant difference in the conventional light treatment after 6 days, and there was no change in the soluble sugar content of Zishan lettuce with the increase in light intensity. There was no change in the soluble sugar content of Zishan lettuce compared with the conventional light treatment after 12 days, and the soluble sugar content of Zishan lettuce increased with the increase in light intensity (Figure 2B).

As shown in Figure 2C, the nitrate content of Yidali lettuce was reduced after 6 days of CL exposure compared to the conventional light treatment, and the nitrate content of Yidali lettuce decreased. The nitrate content of Yidali lettuce decreased after 12 days compared with the conventional light treatment and decreased with increasing light intensity. The nitrate content of Zishan lettuce showed no significant difference in the conventional light treatment after 6 days and decreased with increasing light intensity. The pattern of nitrate content of Zishan lettuce after 6 days was the same as that after 12 days (Figure 2D).

### 3.3. Effects of LED RED–BLUE CL Intensity on Anthocyanin, Total Phenolic and Flavonoid Contents in Two Lettuce Varieties

**Figure 3:**
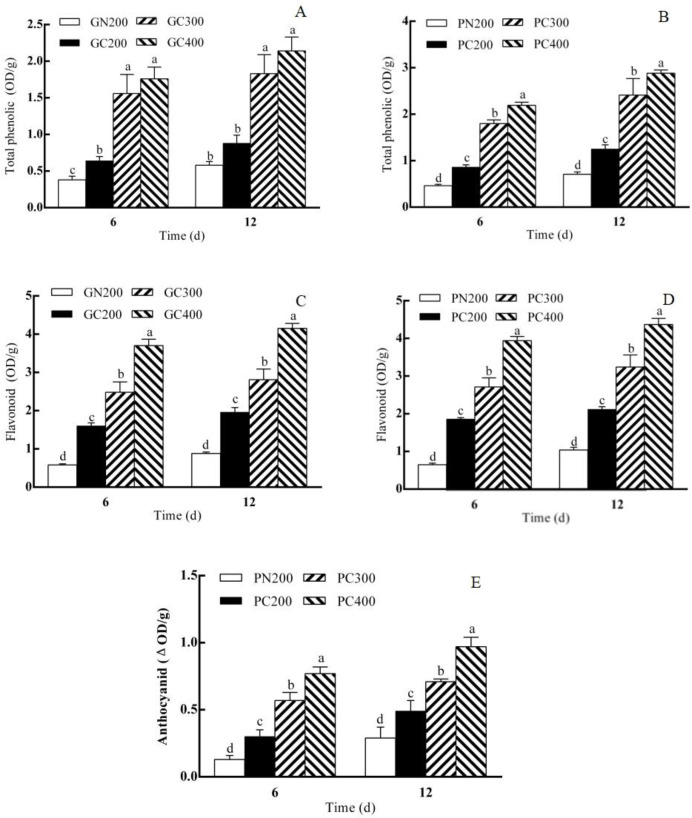
Effect of LED red–blue CL intensities on the contents of antioxidant substances in Yidali (**B**,**D**) and Zishan (**A**,**C**,**E**) lettuces after 6 and 12 days of treatment. Values and bars represent the means of three replicates ± SD. Within a time point, different letters indicate significant differences at the *p* < 0.05 level (Duncan’s multiple range test) over 6 and 12 days of separate tests.

As shown in Figure 3E, the anthocyanin content of Zishan lettuce increased after 6 days of CL compared to the conventional light treatment. The anthocyanin content of Zishan lettuce increased with increasing light intensity after 6 days of treatment. The anthocyanin content of Zishan lettuce after the 12-day treatment was the same as that after 6 days. However, no anthocyanin was detected in the Yidali lettuce.

As shown in Figure 3A, the total phenolic content of both Yidali and Zishan lettuce increased after 6 days of CL compared to the conventional light treatment. The total phenolic content of both lettuce varieties increased with increasing light intensity. There was no difference in the total phenol content of Yidali lettuce compared with the conventional light treatment after 12 days of CL intensity, while the total phenol content of Zishan lettuce increased compared with the conventional light treatment. The total phenol content of both lettuce varieties increased with increasing light intensity (Figure 3B).

As shown in Figure 3C, the flavonoid content of both Yidali lettuce and Zishan lettuce increased after 6 days of CL compared to the conventional light treatment. The total phenolic content of both Yidali and Zishan lettuce increased with increasing light intensity. The pattern of flavonoid content of both lettuce varieties after 12 days of CL treatment was the same as that after 6 days (Figure 3D).

### 3.4. Effects of LED Red–Blue CL Intensity on the DPPH of Two Lettuce Varieties

**Figure 4:**
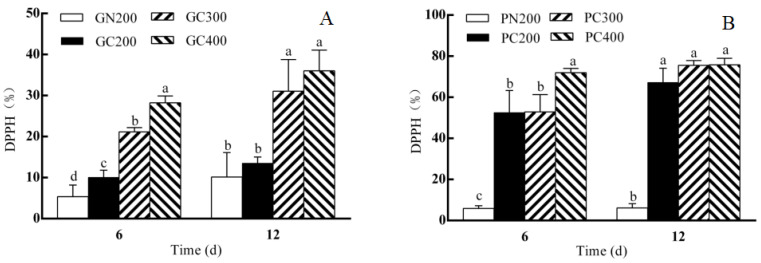
Effect of LED red–blue CL intensities on the DPPH of Yidali (**A**) and Zishan (**B**) lettuce after 6 and 12 days of treatment. Values and bars represent the means of three replicates ± SD. Within a time point, different letters indicate significant differences at the *p* < 0.05 level (Duncan’s multiple range test) over 6 and 12 days of separate tests.

As shown in Figure 4A, the DPPH of Yidali lettuce on the sixth day of CL treatment with the same light intensities increased compared with the conventional light treatment, and the DPPH of Yidali lettuce increased with the increase in light intensity. Compared with the conventional light treatment, the DPPH of Yidali lettuce did not change on the 12th day of CL but increased with the increase in light intensity. On the sixth day of CL treatment with the same light intensities, the DPPH of Zishan lettuce increased compared with the conventional light treatment, and the DPPH of Zishan lettuce increased with the increase in light intensity. Compared with the conventional light treatment, DPPH increased on the 12th day of CL and remained unchanged with the increase in light intensity (Figure 4B).

### 3.5. Effects of LED Red–Blue CL Intensities on the MDA, H_2_O_2_ and Superoxide Anion Contents of Two Lettuce Varieties

**Figure 5:**
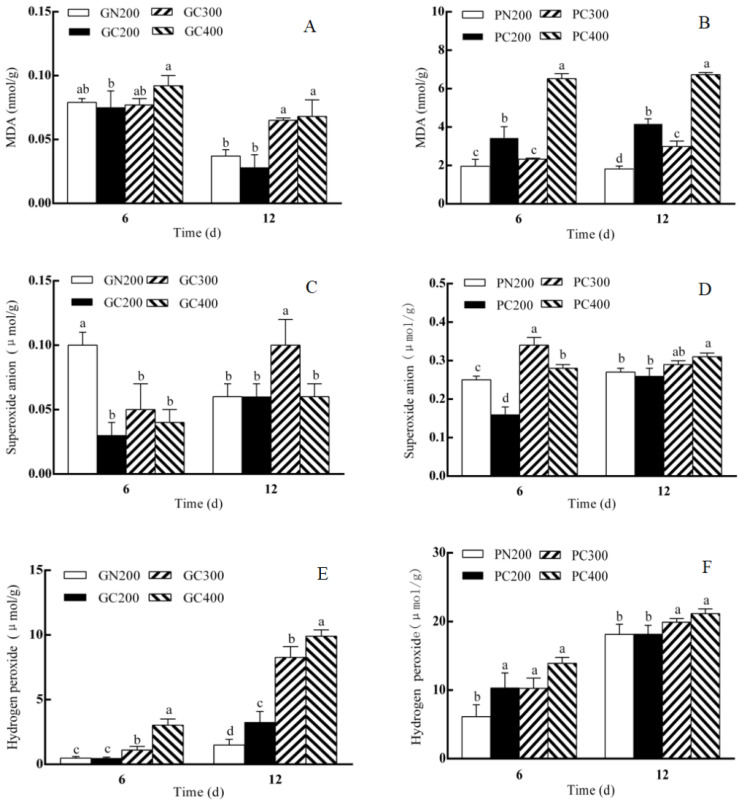
Effect of LED red–blue CL intensities on the MDA, hydrogen peroxide and superoxide anion contents of Yidali (**A**,**C**,**E**) and Zishan (**B**,**D**,**F**) lettuces after 6 and 12 days of treatment. Values and bars represent the means of three replicates ± SD. Within a time point, different letters indicate significant differences at the *p* < 0.05 level (Duncan’s multiple range test) over 6 and 12 days of separate tests.

As shown in Figure 5A, the MDA content of Yidali lettuce decreased after 6 days compared to the conventional light treatment and increased with increasing light intensity. Compared with the conventional light treatment, the MDA content of Yidali lettuce was unchanged on the 12th day of CL exposure and increased with increasing light intensity. The MDA content of Zishan lettuce increased after 6 days compared with the conventional light treatment, and it tended to decrease first and then increase with increasing light intensity. The pattern of MDA content of Zishan lettuce after 12 days was the same as that after 6 days (Figure 5B).

As shown in Figure 5C, the superoxide anion content of Yidali lettuce decreased after 6 days compared with the conventional light treatment, and there was no change in the superoxide anion content of Yidali lettuce with the increase in light intensity. The superoxide anion content of Yidali lettuce was not different from the conventional light treatment after 12 days, and the superoxide anion content of Yidali lettuce showed a trend of increasing and then decreasing with the increase in light intensity. The superoxide anion content of Zishan lettuce decreased after 6 days compared with the conventional light treatment, and the superoxide anion content of Zishan lettuce increased first and then decreased with the increase in light intensity. There was no difference in the superoxide anion content of Zishan lettuce compared with the conventional light treatment after 12 days, and the superoxide anion content of Zishan lettuce increased with the increase in light intensity (Figure 5D).

As shown in Figure 5E, compared to the conventional light treatment, the hydrogen peroxide content of Yidali lettuce was unchanged on day 6 of the CL and increased with increasing light intensity. The content of hydrogen peroxide in Yidali lettuce increased on day 12 of CL treatment compared to the conventional light treatment, and the content of hydrogen peroxide in Yidali lettuce increased with the increase in light intensity. Compared to the conventional light treatment, the hydrogen peroxide content of Zishan lettuce increased on day 6 of the CL and remained unchanged with increasing light intensity. The hydrogen peroxide content of Zishan lettuce decreased on the 12th day of CL compared to the conventional light treatment and increased with increasing light intensity (Figure 5F).

## 4. Discussion

### 4.1. Effects of CL Intensity of LED Red–Blue Light on Yield of Two Lettuce Cultivars

Our data showed that the yield of two lettuces exposed to CL tended to increase with light intensity from 200 to 400 μmol·m^−2^·s^−1^, compared to conventional light treatment, at two sampling times, and the highest yield was obtained at 300 or 400 μmol·m^−2^·s^−1^. Some previous studies reported similar results with lettuce as an experimental plant [7]. The reason might be attributed to the photosynthetic duration for lettuce under CL being prolonged, with a shortened dark period. Apparently, a longer photosynthetic duration can produce more photosynthetic carbohydrates and reduce respiration-based consumption. Therefore, CL can be used as a way to increase lettuce productivity in plant factories with LED light. Our results indicated that the leaf area of two lettuces under CL tended to decrease or remain unchanged with light intensity from 200 to 400 μmol·m^−2^·s^−1^ compared to conventional light both at two sampling times, and the lowest values were obtained at 300 or 400 μmol·m^−2^·s^−1^. Usually, a larger leaf area is a pathway to increase lettuce yield [31], while under CL, the leaf area is not a positive physiological mechanism for yield improvement. Therefore, lettuce yield promotion under CL was due to a longer photoperiod rather than leaf area adaptation.

### 4.2. Effects of CL Intensity of LED Red–Blue Light on the Soluble Sugar and Nitrate Contents of Two Lettuce Cultivars

CL significantly increased the soluble sugar content and decreased the nitrate content of both lettuces, which had a great role in enhancing the nutritional quality of lettuces. It has been found that the photosynthetic products of plants significantly increased and the nitrate content significantly decreased under the combined effect of red–blue light [32]. The present study showed that the soluble sugar content of Yidali lettuce increased and the nitrate content decreased in both Yidali and Zishan lettuces with the same light intensity after 6 and 12 days of CL exposure, compared to conventional light. The soluble sugar content of both lettuces remained unchanged and the nitrate content decreased with increasing light intensity after 6 days of CL. The soluble sugar content of both lettuces increased and the nitrate content decreased with increasing light intensity after 12 days of CL, which is the same as the results of Zhou et al. (2012) [14] and Bian et al. (2016) [33]. The effect of the light environment on plant growth is very complex, and some studies have shown that adding green light or changing the red/blue light ratio in CL treatment has a dramatic effect on both plant yield and quality [34]. Therefore, the effects of CL intensity and light-quality changes on plant growth and changes in plant circadian rhythms need to be explored next.

### 4.3. Effects of CL Intensity of LED Red–Blue Light on the DPPH, and MDA, Hydrogen Peroxide and Superoxide Anion Contents of Two Lettuce Varieties

Based on the previous study of Zha et al. (2020) [8], the light intensity parameters and sampling time were improved in this study, the quality and ROS content of the two lettuces were systematically studied and analyzed, and the test indexes were improved. This is the first systematic study on the long-term CL exposure of Zishan lettuce, which has significant practical implications. Arve et al. (2013) [35] found that the dark period was important for plant stomatal development. It is important that the stomata of plants under CL find it difficult to retain water, resulting in lower plant water content. When plants receive more light energy than they can utilize, the excess light energy will react in chloroplasts, leading to the accumulation of ROS. Moreover, the higher the light intensity, the faster the production and accumulation of ROS content [36]. The excessive accumulation of carbohydrates in plant leaves under CL stress led to leaf senescence and the down-regulation of photosynthesis, and the carbohydrate accumulation resulted in the electron acceptor being over-reduced and the electron transport chain binding electrons to O_2_, inducing ROS accumulation [37], resulting in leaf photo-oxidative stress, photosynthetic down-regulation and leaf senescence [38]. In this study, it was found that the superoxide anion contents of the two varieties of lettuce and the MDA content of the Yidali lettuce continuously decreased compared with the conventional light treatment for 6 days. With the increase in light intensity, the content of hydrogen peroxide, MDA and superoxide anions of the two varieties of lettuce were increased, while the contents of MDA and superoxide anions in Zishan lettuce increased first and then decreased. Also, with the increase in light intensity, the content of hydrogen peroxide, MDA of Yidali lettuce and superoxide anions of Zishan increased, and the superoxide anion content of Yidali lettuce first increased first and then decreased. However, the MDA of Zishan lettuce first decreased and then increased. The difference in hydrogen peroxide and superoxide anion content between the two varieties lettuces may be one of the reasons for their different sensitivities to CL. CL could promote the accumulation of hydrogen peroxide and superoxide anion content and increase them with light intensity. The accumulation of ROS was mainly caused by the accumulation of hydrogen peroxide. The accumulation of hydrogen peroxide and superoxide anions in Yidali lettuce was more obvious, but the DPPH in Zishan lettuce was significantly higher than that in Yidali lettuce. Moreover, the hydrogen peroxide and superoxide anion content and DPPH of the two lettuces increased differently with the change of light intensity, which was the cause of injury. With the increase in light intensity, the increase in hydrogen peroxide, superoxide anions and MDA content in Yidali lettuce was much higher than that in Zishan lettuce, but the increase in DPPH was less than that in Zishan leaves, indicating that a large amount of hydrogen peroxide and superoxide anions accumulated, but the scavenging ability was not improved accordingly. The removal of ROS in plants is accomplished by enzymatic reactions and antioxidant systems. Superoxide anions and hydroxyl radicals are ROS in the form of free radicals, while hydrogen peroxide and singlet oxygen are ROS in the form of non-free radicals. Compared with other ROS, hydrogen peroxide has a relatively long survival cycle and high stability, and it is not easy to decompose and remove. Superoxide anions are the initial ROS produced in the process of hydrogen peroxide production, which has a short survival cycle and is easy to remove. Therefore, increasing DPPH can effectively remove superoxide anions from lettuce, but it has little effect on hydrogen peroxide content, which was found in the results of this study. The change of ROS content and the accumulation of carbohydrates may be the reasons for the differences in CL tolerance between the two lettuces. Our previous study found that the change of circadian rhythm may also lead to a certain degree of leafy damage to the plants [39], and the specific mechanism needs to be further explored. Comparatively, the Zishan cultivar had higher ROS and MDA contents and DPPH (1 to 100 times) than the Yidali cultivar under high-intensity CL. The high CL tolerance of Zishan was attributed to stronger DPPH.

### 4.4. Effects of CL Intensity of LED Red–Blue Light on the Contents of Antioxidant Substances in Two Lettuce Cultivars

In this study, we found that the antioxidant content in both lettuces, except anthocyanins, was the same or increased under the same light intensity after 6 days and increased with increased light intensity, and the pattern was the same after 12 days of CL exposure as after 6 days. The absence of physiological damage in Zishan lettuce may be related to its richness in anthocyanins, total phenols and flavonoids, and the antioxidant system may be involved in the CL-tolerant mechanism of Zishan lettuce, which needs to be further investigated. The fact that Zishan lettuce was rich in anthocyanins but Yidali lettuce does not contain anthocyanin may also be a major reason why the two lettuces have very different tolerance capacities to CL. Zishan lettuce was rich in antioxidant substances and had strong antioxidant capacity, indicating that the nutritional quality of Zishan lettuce is higher than that of Yidali lettuce. Velez-Ramirez et al. (2017) [40] hypothesized that the circadian rhythm asynchrony caused by the CL was the main factor in CL-induced stress in plants. CL-sustained photosynthetic energy supply leads to leaf carbohydrates, and the excessive accumulation of ROS induces photo-oxidative stress. The current data showed similar results.

The regulation of LED red–blue CL light intensity can improve the yield and nutritional quality of Yidali and Zishan lettuces. For Zishan lettuce, high-intensity LED red–blue CL was beneficial for obtaining higher yields and quality. However, for Yidali lettuce, low light intensity (≤200 μmol·m^−2^·s^−1^) and an irradiation duration less than 12 days can be used to improve the yield and quality of LED red–blue CL treatment. However, a high-intensity LED red–blue CL of long duration can cause leaf injury symptoms and reduce the appearance quality of Yidali lettuce. Employing LED red–blue CL is a good method to effectively improve the nutritional quality of hydroponic lettuce. Comparatively, the Zishan cultivar contained higher contents than the Yidali cultivar under high-intensity CL. The high CL tolerance of Zishan might be attributed to its stronger antioxidant capacity, as a result of more antioxidant substances. It can be inferred from this study that purple-leaf lettuces (and even other leafy vegetable species) may be CL-tolerant plants due to their strong antioxidant capacity, provided by the antioxidant system. To sum up, the current findings be applied to optimize LED red–blue CL application strategies in commercial settings, especially for similar crops. We believe that LED red–blue CL application can be used in plant factories with LED lighting and vertical farming, using hydroponics technology on increasing numbers of plant species and cultivars.

## 5. Conclusions

The dry and fresh shoot weight of two lettuces exposed to red–blue CL tended to increase with light intensity from 200 to 400 μmol·m^−2^·s^−1^ compared to conventional light treatment, while the leaf area tended to decrease or remained unchanged. High-intensity (300 and 400 μmol·m^−2^·s^−1^) CL could increase the soluble sugar and antioxidant substance (anthocyanins, flavonoids and total phenols) content while reducing the nitrate content of two lettuces. Also, high-intensity CL could significantly increase the malondialdehyde, hydrogen peroxide and superoxide anion contents and DPPH of two lettuces. Similar results were found at 6 and 12 days after light treatment. Comparatively, the Zishan cultivar contained a higher amount of antioxidant substances, ROS, MDA content and DPPH (1 to 100 times) than the Yidali cultivar under high-intensity CL. In summary, high-intensity red–blue CL could improve the yield and nutritional value of both Yidali and Zishan lettuces. The high CL tolerance of Zishan was attributed to stronger antioxidant capacity, brought about by a greater content of antioxidant substances and DPPH, while the accumulation of ROS and antioxidant substances might interact.

## Figures and Tables

**Table 1:** Effect of LED red–blue CL intensities on yield and leaf area of two lettuces after 6 and 12 days of treatment.

Sampling Times	Treatments	Shoot Fresh Weight (g)	Shoot Dry Weight (g)	Leaf Area (cm^2^)
6 days	GN200	26.75 ± 2.37 b	1.56 ± 0.13 b	520 ± 32.85 a
	GC200	29.33 ± 1.69 b	1.84 ± 0.09 b	474 ± 11.66 a
	GC300	37.89 ± 3.38 a	2.69 ± 0.20 a	499 ± 48.51 a
	GC400	32.25 ± 3.14 ab	2.67 ± 0.12 a	394 ± 14.73 b
	PN200	10.50 ± 1.39 b	0.97 ± 0.13 b	280 ± 22.00 a
	PC200	12.17 ± 0.75 b	1.16 ± 0.04 ab	286 ± 17.05 a
	PC300	15.61 ± 2.09 a	1.42 ± 0.32 a	246 ± 20.73 ab
	PC400	11.09 ± 0.41 b	1.19 ± 0.05 ab	228 ± 13.49 b
12 days	GN200	52.37 ± 6.08 b	3.88 ± 0.09 b	1082 ± 31.03 a
	GC200	57.29 ± 8.74 ab	4.20 ± 0.26 b	950 ± 111.46 a
	GC300	63.02 ± 3.43 ab	5.00 ± 0.20 a	673 ± 28.45 b
	GC400	69.28 ± 8.97 a	5.19 ± 0.70 a	653 ± 58.51 b
	PN200	28.06 ± 0.29 c	3.33 ± 0.35 b	675 ± 93.65 a
	PC200	31.12 ± 0.83 bc	3.58 ± 0.30 b	678 ± 35.32 a
	PC300	35.21 ± 0.31 ab	3.95 ± 0.10 b	622 ± 22.38 a
	PC400	40.05 ± 6.71 a	4.82 ± 0.39 a	623 ± 37.64 a

Note: GN and GC stand for green-leaf Yidali lettuce under conventional light and continuous light, while PN and PC stand for purple-leaf Zishan lettuce under conventional light and continuous light. The numbers along with GN, GC, PN and PC are light intensity of conventional light and CL. The significance of the two lettuces was tested on day 6 and day 12, respectively. The Lowercase letters followed the data represent significant differences *p* < 0.05 between treatments for the same lettuce cultivar at the same sampling time.

## Data Availability

The data are contained within the article.
